# Supplementary material for: Isomerization of Asp7 in Beta-Amyloid Enhances Inhibition of the α7 Nicotinic Receptor and Promotes Neurotoxicity
Source: Cells. 2019 Jul 25;8(8):771. doi: 10.3390/cells8080771 (PMC6721525; doi:10.3390/cells8080771)
Supplement: Supplementary file 1 [file cells-08-00771-s001.pdf]

Article

Isomerization of Asp7 in Beta-Amyloid Enhances Inhibition of the α7 Nicotinic Receptor and Promotes Neurotoxicity

Evgeny P. Barykin [1], Alexandra I. Garifulina [2], Elena V. Kruykova [2], Ekaterina N. Spirova [2], Anastasia A. Anashkina [1], Alexei A. Adzhubei [1], Irina V. Shelukhina [2], Igor E. Kasheverov [2,3], Vladimir A. Mitkevich [1], Sergey A. Kozin [1], Michael Hollmann [4], Victor I. Tsetlin [2] and Alexander A. Makarov [1,*]

Supplementary Methods

Cytochemical staining of N2a cells

To assess cell expression of human α7 nAChR, the transfected and non-transfected N2a cells plated on glass coverslips were stained with Alexa-Fluor 555-conjugated α-bungarotoxin (50 nM) for 20 min at room temperature. Cells were washed extensively with a buffer containing 140 mM NaCl, 2 mM $CaCl_2$, 2.8 mM KCl, 4 mM $MgCl_2$, 20 mM HEPES, 10 mM glucose; pH 7.4 to remove any unbound toxin. Fluorescent staining was observed with an epifluorescence microscope (Olympus, Japan). Pictures were taken and processed with CellA Imaging Software (Olympus Soft Imaging Solutions GmbH, Germany) and open-source applications CellX and Image J.

Photo-induced cross-linking of amyloid peptides

Synthetic peptides $A\beta_{42}$ and iso-$A\beta_{42}$ (Biopeptides) were treated with hexafluoroisopropanol, dried, and dissolved in 10 mM NaOH at concentration of 0.5 mM. Peptides were brought to 50 µM with 10 mM Na-phosphate buffer (pH 7.2). To prepare a reaction mix, 18 µl aliquot of 50 µM $A\beta_{42}$ or iso-$A\beta_{42}$ was transferred to 200 µl PCR tube and 1 µl of 20 mM ammonium persulfate and 1 µl of 1 mM Tris(2,2-bipyridyl)dichlororuthenium(II) hexahydrate. (RuBpy) were added. The reaction mixture was placed to the illumination chamber (100 W incandescent lamp) and irradiated for 3 s. Residual RuBpy was quenched immediately by mixing with 1 µl of 1M dithiothreitol (Sigma) and 20 µl of 2x SDS-PAGE Sample Buffer (Novex). Cross-linked samples were heated for 5 min at 95°C and analyzed with SDS-PAGE in a gradient 6-20% gel.

Supplementary Figures

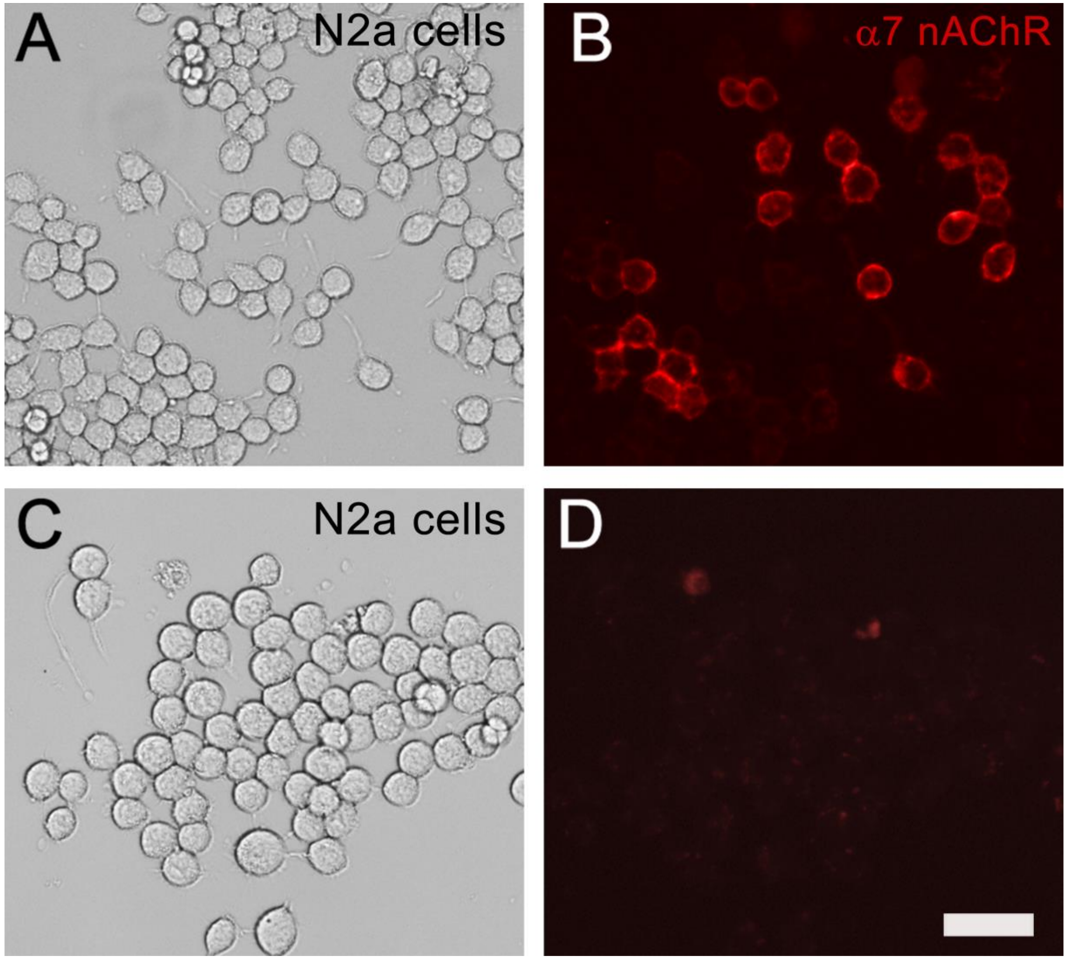

Figure S1. Cytochemical staining of (A, B) N2a cells transfected with plasmids coding human *α*7nAChR, chaperone NACHO and calcium sensor Case12 and (C, D) of non-transfected cells with 50 nM Alexa Fluor 555-*α*-bungarotoxin (*red*). Scale bar, 50 μm.

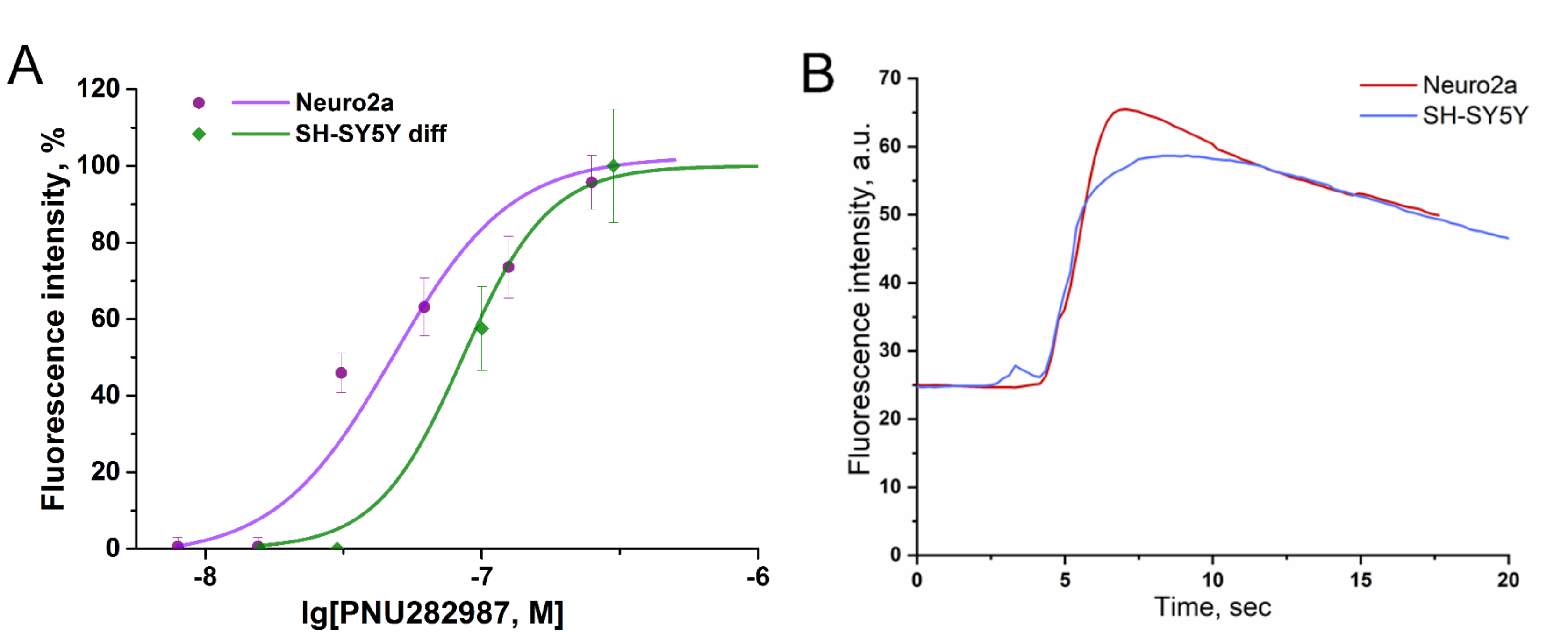

Figure S2. (A) Dose-dependent intracellular calcium rise induced by PNU-282897 stimulation in the α7nAChR-transfected Neuro2a and in differentiated SH-SY5Y (SH-SY5Y diff) cell lines. (B) Kinetics of the α7nAChR-mediated intracellular Ca^{2+}single-cell response to PNU282987 (500nM) stimulation in Neuro2a cell expressing the fluorescent Ca^{2+} sensor Case12 and inSH-SY5Y cell, loaded with a fluorescent dye Fluo-4. The cells were pre-incubated with 10 μM of the α7nAChR positive allosteric modulator PNU120596for 20 min before the agonist application. a.u. – arbitrary units.

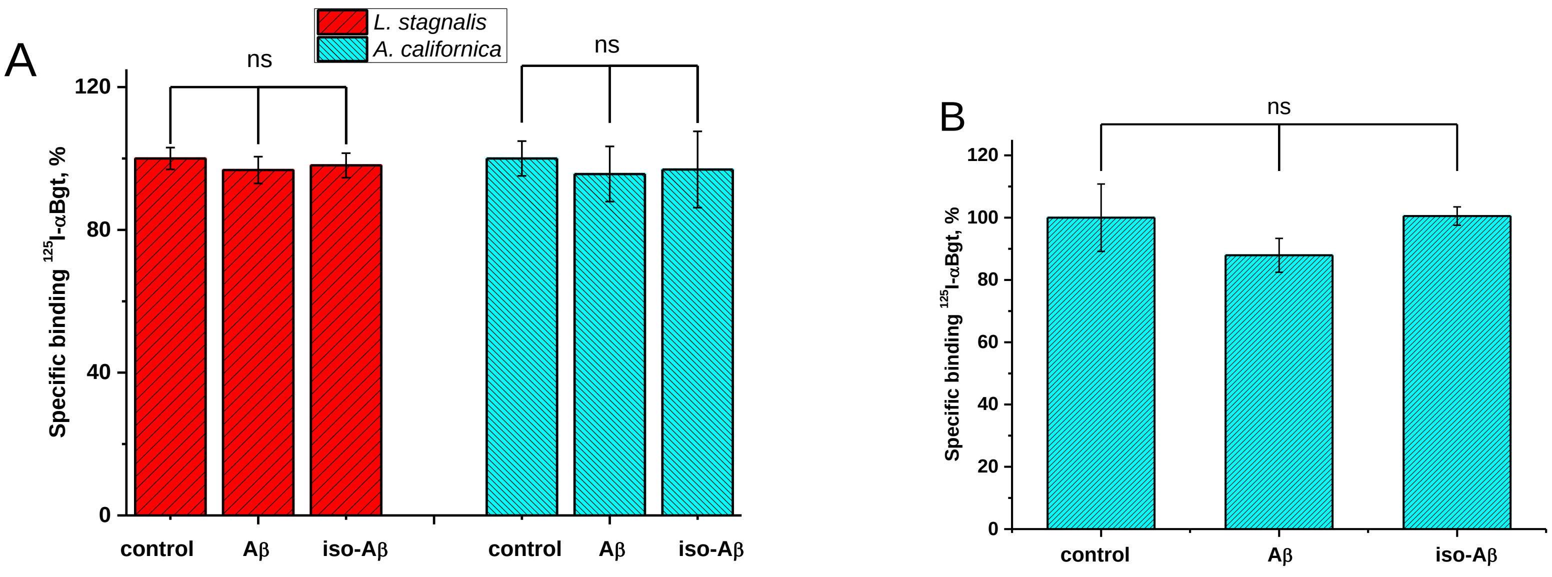

Figure S3**.** Analysis of $A\beta_{42}$ and iso-$A\beta_{42}$ competition with radioiodinated α-bungarotoxin (^{125}I-αBgt) for binding to (A) the acetylcholine-binding protein (AChBP) from *L. stagnalis* (left panel) or *A. californica* (right panel) and (B) to the α7nAChR of GH4C1 cells. Both amyloid peptides (15 μM) were dissolved in DMSO and pre-incubated with the receptor protein for 40 min before ^{125}I-αBgt addition. Specific binding in the control is set to 100%. Data are presented as the mean ± SEM of two experiments with four or five replicates for each point. ns = non-significant.

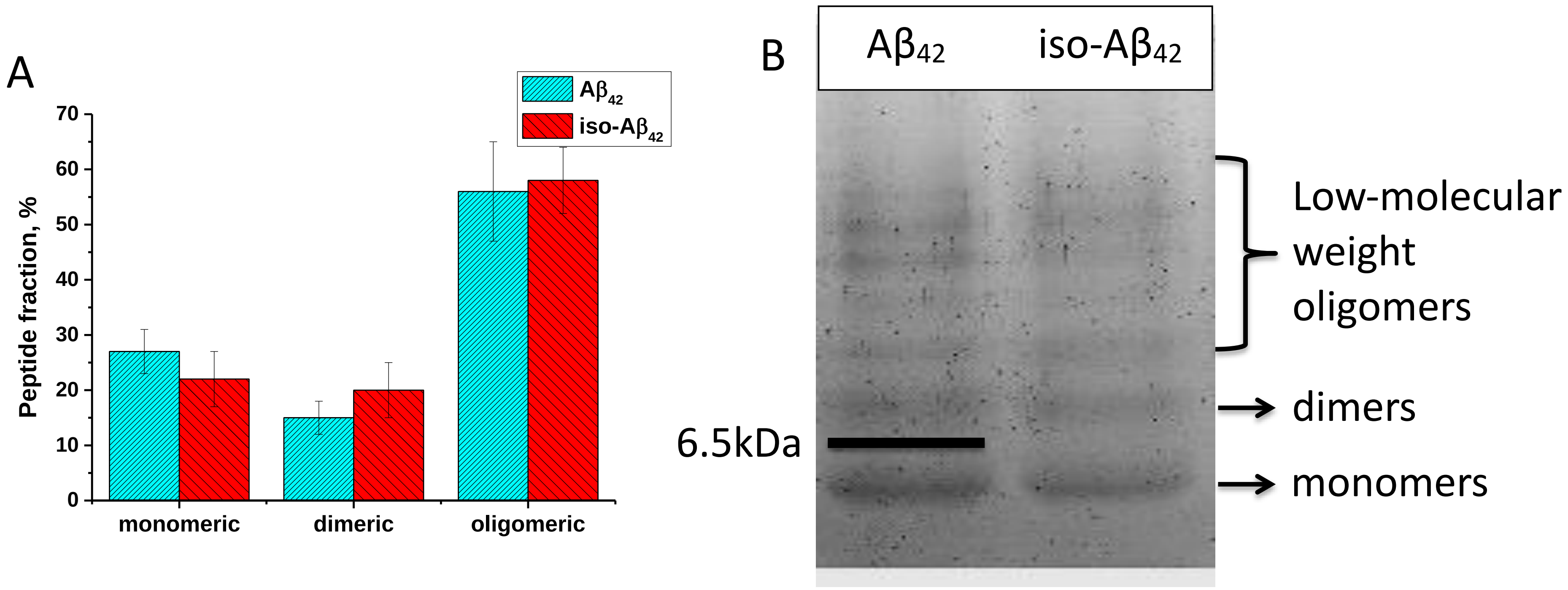

Figure S4.The relative amount of $A\beta_{42}$ and iso-$A\beta_{42}$ peptides in various aggregation states, determined by photo-induced chemical cross-linking. A solution of the peptide in 10 mM Na-phosphate buffer (pH 7.2) was irradiated with visible light. The peptide concentration was 50 μM. Panel A: The proportion of the peptides in monomeric, dimeric and oligomeric (low-molecular weight oligomers) forms. The mean values and standard deviations for three experiments are shown.Panel B: SDS-PAGE analysis of $A\beta_{42}$ and iso-$A\beta_{42}$ peptides' solutions after photo-induced cross-linking. Black bar on the gel represents the position of the 6.5 kDa marker.6-20% gradient gel was used for the separation of the peptides.

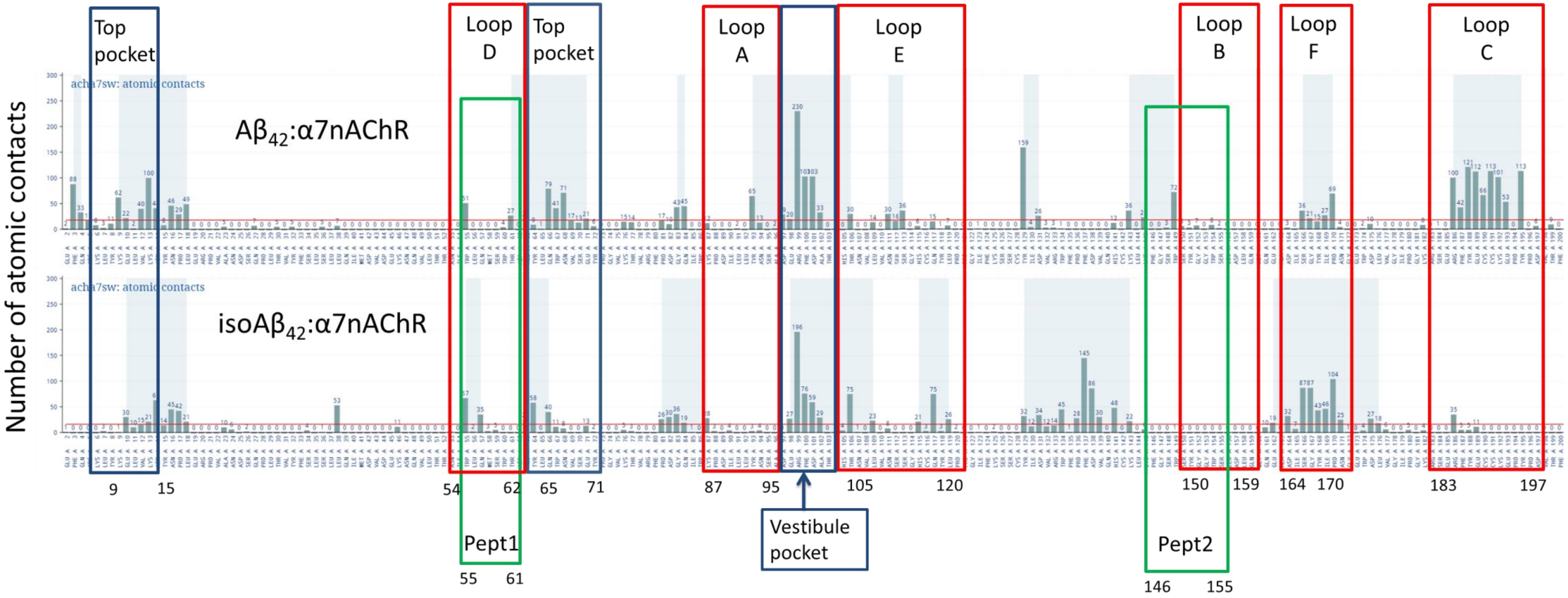

Figure S5. The probability of binding of Aβ_{42} (upper part) and iso-Aβ_{42} (lower part) peptides to amino acid residues of extracellular part of the α7nAChR. Horizontally represented areamino acid residues 2-200 of the extracellular part of the α7nAChR, vertically - the total number of atomic contacts of peptides in all models with each residue. The framework identifies segments of the α7nAChR sequence corresponding to the experimentally established functional sites: Top pocket, Vestibule pocket - allosteric binding sites; Loop A, B, C, D, E, F - loops that form the ligand-binding pocket; Pept1, Pept2 - binding sites for peptides – inhibitors of Aβ interaction with the α7nAChR.

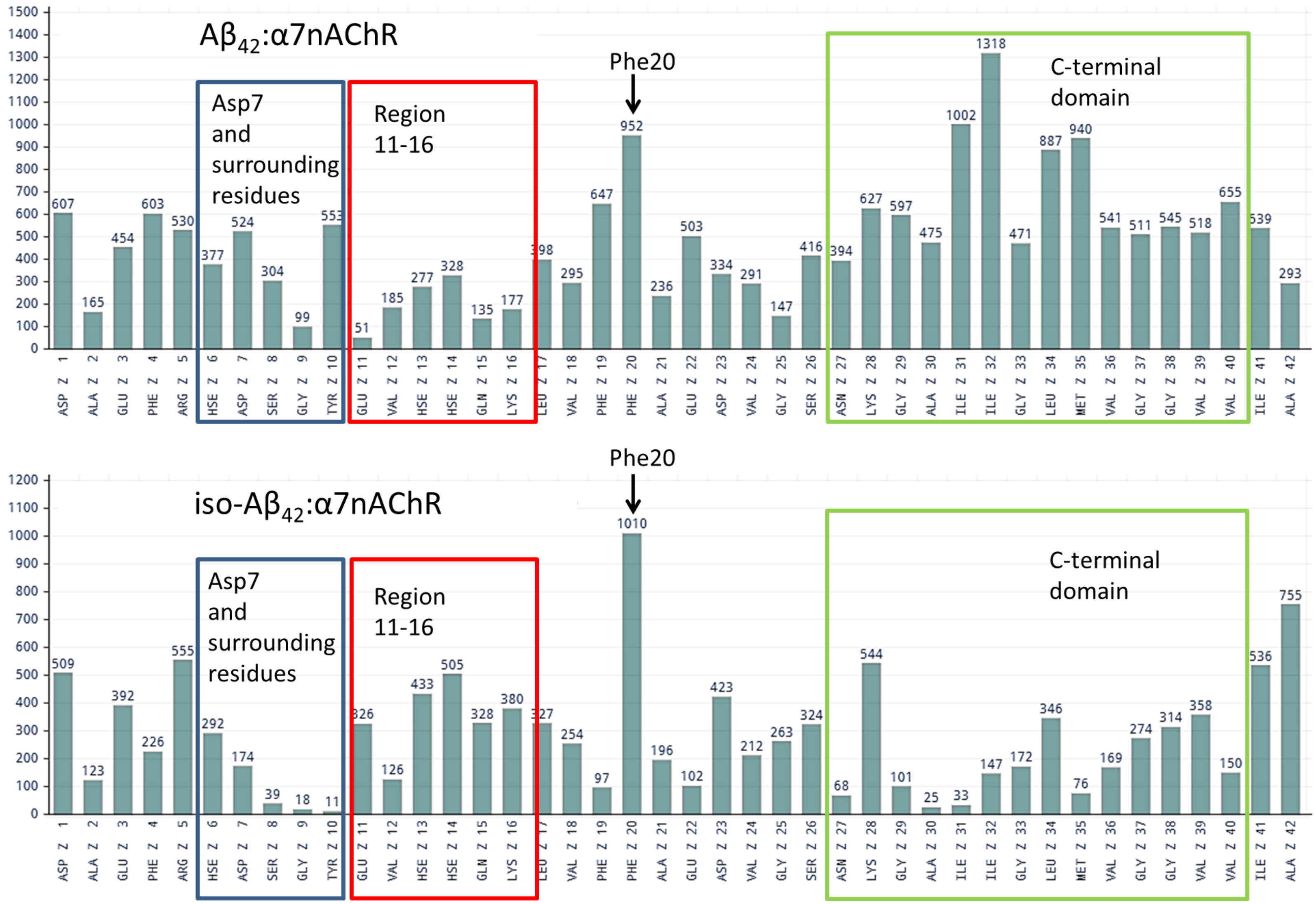

Figure S6. The probability of amino acid residues binding of $Aβ_{42}$ (upper panel) and iso-$Aβ_{42}$ (lower panel) peptides to the extracellular part of the α7nAChR. Horizontally represented are amino acid residues 1-42 of amyloid peptides, vertically - the total number of atomic contacts of a particular residue with the extracellular part of the α7nAChR in all models. The framework highlights the segments of $Aβ_{42}$ or iso$Aβ_{42}$ sequences that bind differently to the α7nAChR.
